# Immunotactoid Glomerulopathy in a Patient With HIV and Anti-hepatitis C Virus (HCV) Positivity: A Unique Clinical Entity

**DOI:** 10.7759/cureus.90015

**Published:** 2025-08-13

**Authors:** Abhishesh Wagle, Rayana Shrestha, Nidhi Varma

**Affiliations:** 1 Nephrology, Albert Einstein College of Medicine, Jacobi Medical Center, Bronx, USA; 2 Department of Internal Medicine, Institute of Medicine, Maharajgunj, Kathmandu, NPL

**Keywords:** glomerulonephritis, hcv and kidney, hiv aids and kidney, immunotactoid glomerulopathy, sodium-glucose cotransporter-2 (sglt2) inhibitors

## Abstract

Immunotactoid glomerulopathy (ITG) is a rare glomerular disease characterized by organized immunoglobulin deposits forming microtubular structures on electron microscopy. While ITG has been individually associated with HIV and hepatitis C virus (HCV) infections, there are few case reports with concurrent HIV infection and anti-HCV antibody positivity.

We report a 57-year-old Hispanic female with HIV infection since 2007 and positive anti-HCV antibodies who presented with nephrotic-range proteinuria (urine protein-to-creatinine ratio: 7,834 mg/gm), hematuria, and hypertension. Renal biopsy revealed enlarged glomeruli with diffuse mesangial expansion, segmental endocapillary proliferation, and characteristic organized microtubular deposits on electron microscopy. Immunofluorescence demonstrated granular staining for IgG (both IgG1 and IgG2 subclasses), kappa and lambda light chains, and C3, confirming polyclonal ITG. Extensive hematologic workup was negative for lymphoproliferative disorders.

Given the patient's immunocompromised status, immunosuppressive therapy was avoided. Treatment focused on optimizing antiretroviral therapy, transitioning from oral to long-acting injectable regimens (including lenacapavir), which improved viral suppression and CD4 counts. Proteinuria management included angiotensin-converting enzyme inhibitors and sodium-glucose cotransporter-2 (SGLT2) inhibitors, resulting in significant proteinuria reduction while maintaining preserved renal function (serum creatinine ≤1.0 mg/dL).

This is a unique case of ITG in a patient with concurrent HIV infection and anti-HCV seropositivity. Our case demonstrates that polyclonal ITG in immunocompromised patients can be effectively managed without immunosuppression through optimized antiretroviral therapy and nephroprotective agents, including SGLT2 inhibitors. This approach offers a novel therapeutic strategy that avoids the risks of immunosuppression in vulnerable populations while achieving favorable clinical outcomes.

## Introduction

Immunotactoid glomerulopathy (ITG) is an uncommon glomerular pathology defined by the presence of microtubular deposits of immunoglobulins visible on electron microscopy. These tactoids are Congo red negative and typically measure 14-60nm in diameter. ITG accounts for 0.04% of native kidney biopsies [1-3]. The deposits are usually composed of IgG, especially the IgG1 and IgG2 subclasses, and may show kappa or lambda light chain restriction [4].

ITG can be monoclonal or polyclonal. Monoclonal cases are often linked with underlying lymphoproliferative diseases, such as chronic lymphocytic leukemia or multiple myeloma. Polyclonal ITG, while less common, is associated with autoimmune conditions and chronic infections including hepatitis C virus (HCV), human immunodeficiency virus (HIV), and occasionally mycobacterial infections [5,6].

The understanding of ITG has evolved significantly through several key studies. Nasr et al. conducted an early clinicopathologic and proteomic study in 2012, which provided important insights into the pathophysiology and clinical characteristics of ITG [4]. This foundational work was later expanded by the same group in 2021, providing one of the most comprehensive analyses of ITG, detailing both clinical and pathological features in a large case series of 73 patients and highlighting the importance of distinguishing between monoclonal and polyclonal patterns using proteomic techniques [5]. Their study reinforced the value of identifying clonality, as treatment strategies vary substantially based on the classification. Monoclonal ITG may benefit from clone-directed therapies, while polyclonal cases should prompt investigation for underlying infection or autoimmune disease. Javaugue et al. contributed to the literature by documenting the long-term renal outcomes in ITG and identifying predictors of progression, emphasizing that timely diagnosis and targeted management improve prognosis [7].

Haas et al. reported three cases of fibrillary/immunotactoid glomerulonephritis in HIV-positive patients, representing the first systematic description of this association [8]. van Biljon et al. described immunotactoid glomerulonephritis in a pediatric HIV patient, highlighting the disease's occurrence across age groups [9].

Similarly Markowitz et al. conducted a landmark study examining four cases of fibrillary glomerulonephritis and two cases of immunotactoid glomerulopathy in association with HCV infection, establishing the clear link between HCV and these fibrillary glomerular diseases [10].

Our case here describes ITG in a patient with both HIV and anti-HCV antibody positivity, highlighting a unique intersection of pathophysiological factors.

## Case presentation

A 57-year-old Hispanic female with known HIV infection, intermittent antiretroviral compliance, and positive anti-HCV antibodies with undetectable HCV RNA was admitted with nephrotic proteinuria (urine protein-creatinine ratio (UPCR)=7834 mg/gm), hematuria, and hypertension (blood pressure=171/75 mmHg). Renal biopsy revealed enlarged glomeruli with diffuse mesangial expansion, segmental endocapillary proliferation, global electron-dense deposits, and characteristic organized microtubular structures. Immunofluorescence showed granular staining for IgG, kappa, lambda chains, and C3, confirming polyclonality.

**Figure 1:**
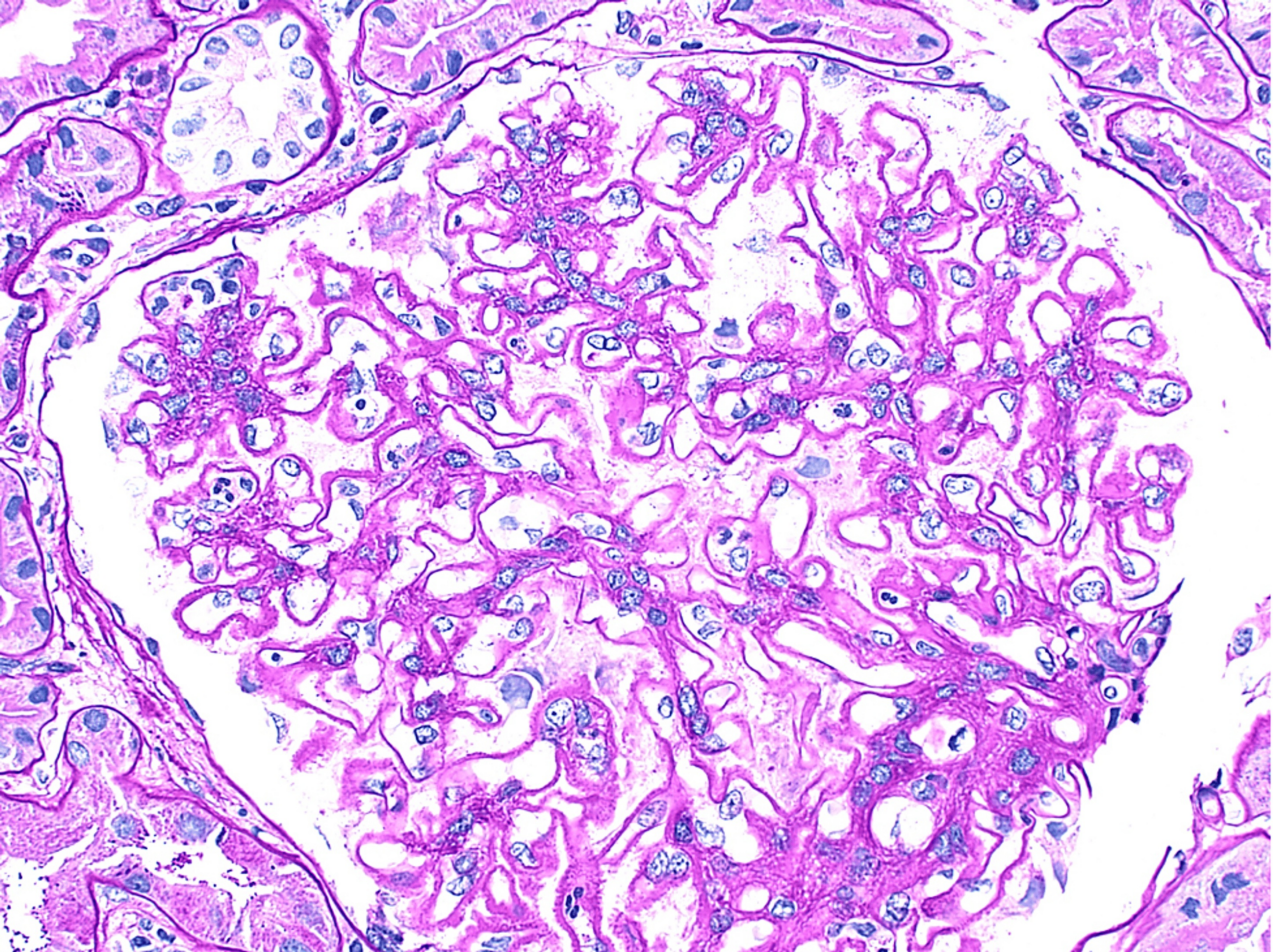
H & E stain of the glomerulus showing enlarged glomeruli with diffuse mild to moderate, segmental to global mesangial expansion of cells and mesangial matrix focally containing glassy eosinophilic immune type deposits

**Figure 2:**
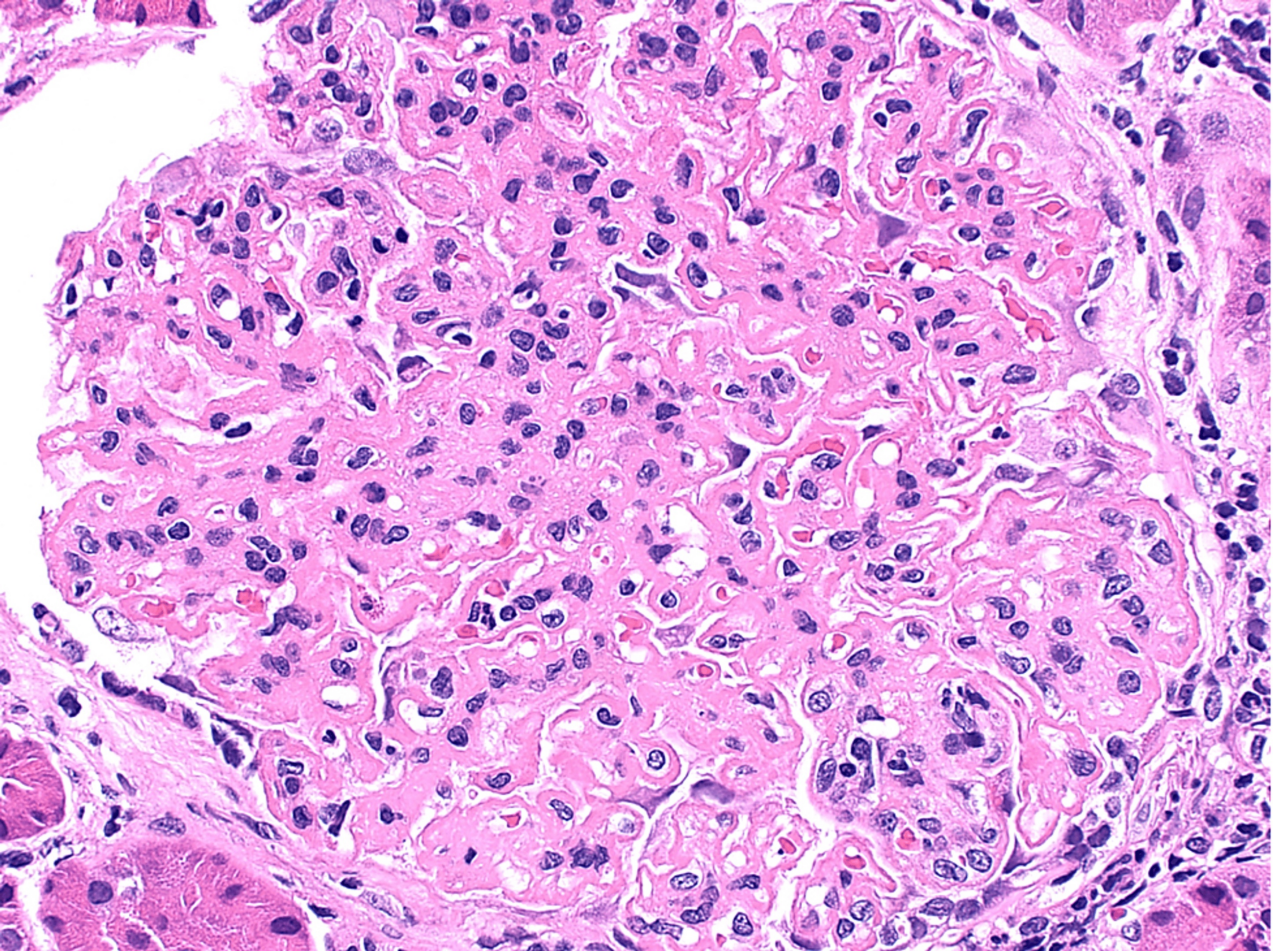
Showing glomerulus with endocapillary proliferation by infiltrating mononuclear leucocytes and neutrophils

**Figure 3:**
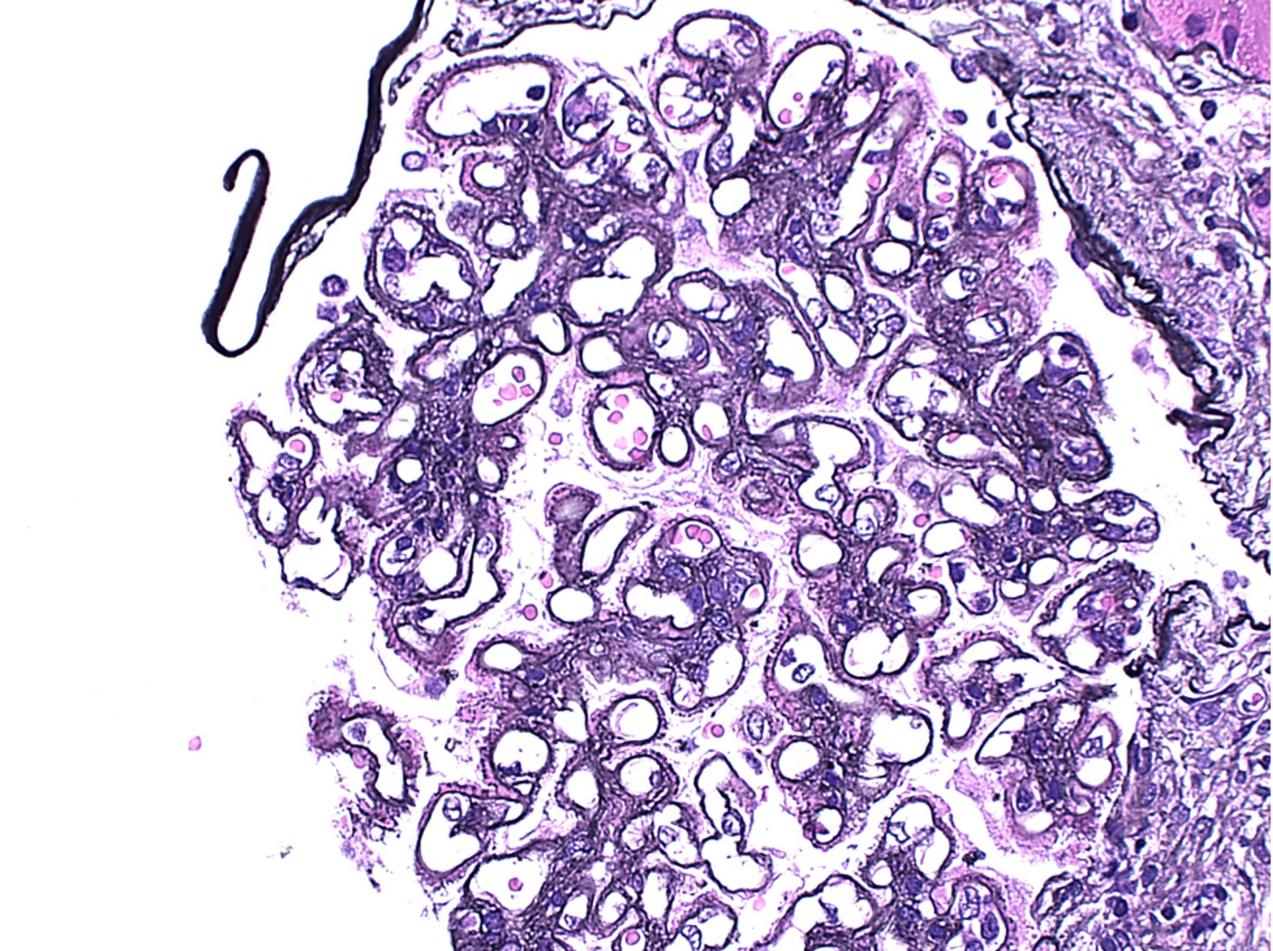
Jones methenamine stain (JMS) of the glomerulus showing diffusely thickened glomerular basement membranes (GBM) owing to presence of subepithelial fuchsinophilic immune deposits associated with intervening spikes

Light microscopy demonstrated enlarged glomeruli with diffuse mild to moderate, segmental to global mesangial expansion of cells and mesangial matrix focally containing glassy eosinophilic immune type deposits (Figure 1), with approximately 25% of glomeruli showing segmental endocapillary proliferation by infiltrating mononuclear leucocytes and neutrophils (Figure 2). Glomerular basement membranes (GBM) were diffusely thickened owing to presence of subepithelial fuchsinophilic immune deposits associated with intervening spikes (Figure 3).

**Figure 4:**
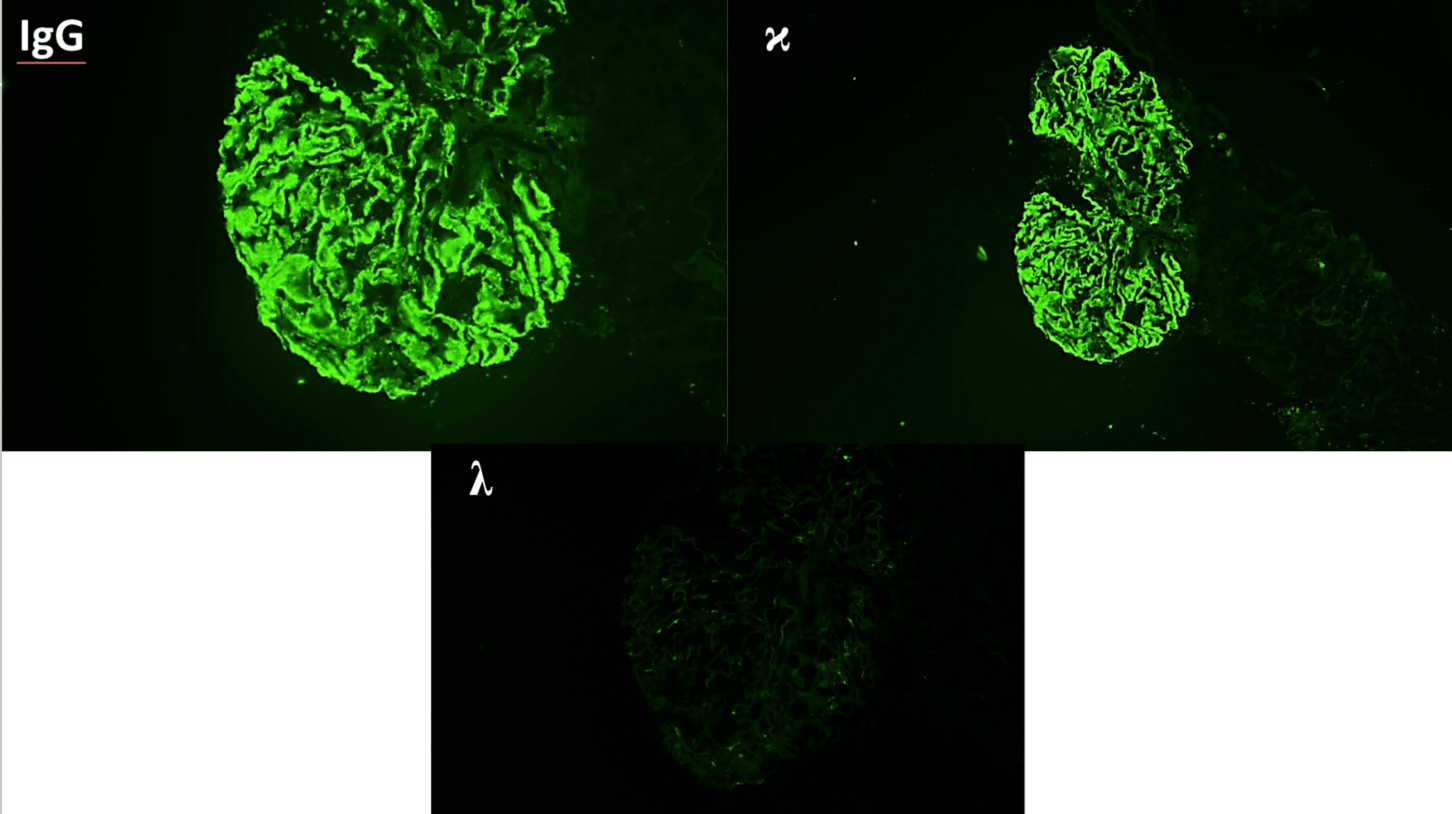
Immunofluorescence revealed 3+ granular global mesangial and global glomerular capillary wall staining for IgG and kappa.

**Figure 5:**
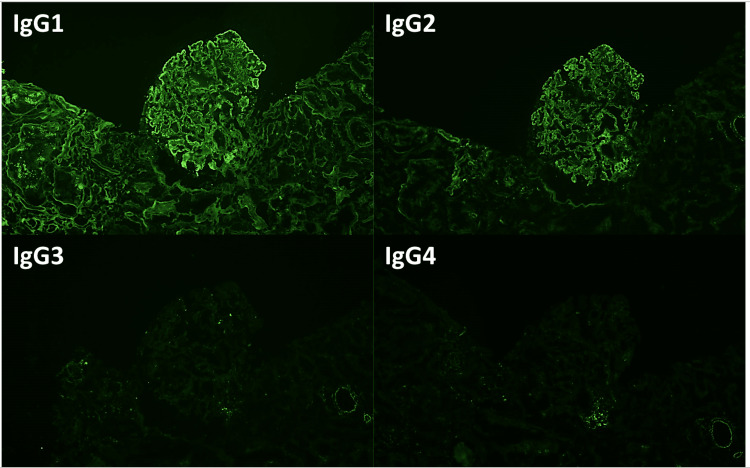
Immunofluorescence showing staining for IgG1 and IgG2

**Figure 6:**
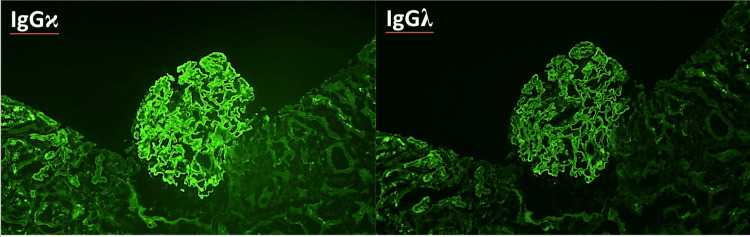
Immunofluorescence showing staining for IgG kappa and IgG gamma.

Immunofluorescence revealed 3+ granular global mesangial and global glomerular capillary wall staining for IgG and kappa with 2+ staining (Figure 4). IgG subtype and IgG heavy and light chain immunofluorescence revealed staining for IgG1 and IgG2 (Figure 5) and both IgG-kappa and IgG-lambda (Figure 6).

**Figure 7:**
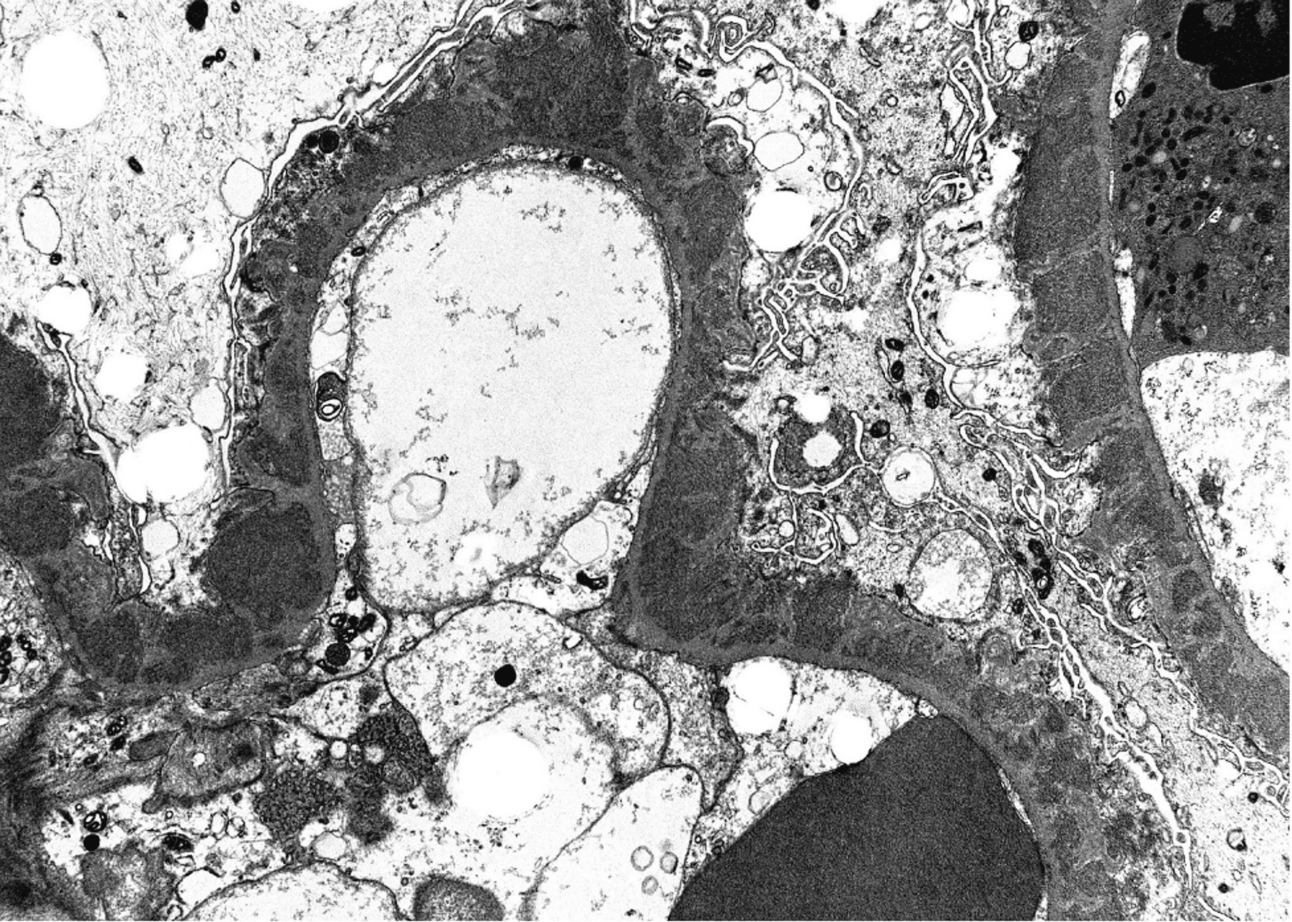
Electron microscopy showing global subepithelial deposits and scattered segmental subendothelial deposits.

**Figure 8:**
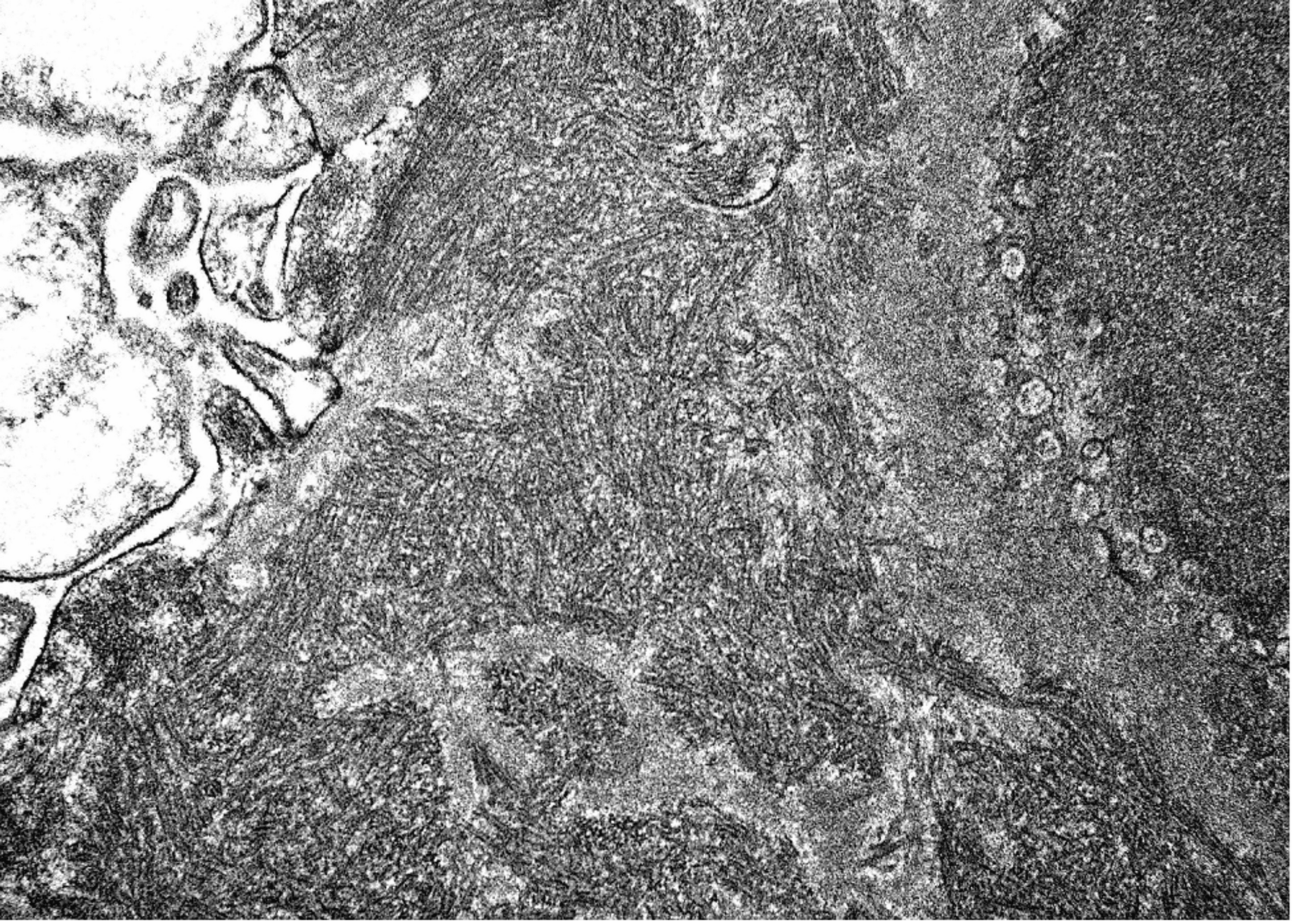
Electron microscopy showing organized substructure, forming microtubules with hollow core in parallel alignment.

Electron microscopy showed mesangial areas expanded by cells and matrix containing global electron dense deposits, global subepithelial deposits and scattered segmental subendothelial deposits (Figure 7). These deposits displayed organized substructure, forming microtubules with a hollow core in parallel alignment (Figure 8).

Treatment and management

The patient's management was guided by the dual challenge of underlying HIV infection and histological diagnosis of ITG. Given the polyclonal nature of the deposits and absence of any identifiable lymphoproliferative disorder on extensive workup, immunosuppressive therapy was avoided. Immunosuppression in such patients carries considerable risk due to the potential for opportunistic infections.

Optimal medication strategies were achieved through adjustments from oral therapies such as (bictegravir/emtricitabine/tenofovir alafenamide)/(dolutegravir/rilpivirine) to long-acting injectable options including cabotegravir and rilpivirine, and eventually lenacapavir, which improved her virological suppression and absolute CD4 count.

Concurrently, lisinopril was added to reduce proteinuria and had to be titrated carefully due to episodes of hypotension and hyperkalemia. After SGLT2 inhibitors were initiated, there was a subsequent reduction in proteinuria. The patient's renal function remained preserved throughout, with serum creatinine mostly ≤1.0 mg/dL.

Table 1 shows the timeline, intervention and key characteristics.

**Table 1:** Timeline of renal function (serum creatinine in mg/dl), proteinuria (mg/gm), HIV RNA viral load (copies/ml), CD4 count (cells/microL) and key interventions.

^Timeline^	^Serum Creatinine (mg/dL)^	^Proteinuria (mg/gm)^	^Viral Load(copies/ml)^	^CD4 Count( Absolute CD4 count in cells/microL)^	^Treatments Initiated^
^Baseline^	^-^	^-^	^136,673^	^-^	^-^
^Month 2^	^0.7^	^1059^	^1485^	^153^	^Bictegravir/emtricitabine/tenofovir alafenamide^
^Month 14^	^-^	^1317^	^6503^	^201^	^-^
^Month 19^	^-^	^1620^	^25,772^	^121^	^-^
^Month 23^	^-^	^1870^	^93,818^	^134^	^-^
^Month 39^	^1.2^	^7834^	^10,300^	^77^	^Hospitalized^
^Month 41^	^0.8^	^5990^	^-^	^106^	^Renal biopsy^
^Month 43^	^1^	^4643^	^<20^	^93^	^Lisinopril started^
^Month 46^	^0.8^	^7783^	^117^	^98^	^Cabotegravir^
^Month 51^	^-^	^3504^	^969^	^115^	^Lenacapavir^
^Month 54^	^0.8^	^4555^	^<20^	^149^	^-^
^Month 58^	^0.9^	^2412^	^-^	^29^	^-^
^Month 63^	^-^	^-^	^-^	^-^	^Started on SGLT2i^
^Month 65^	^1^	^1543^	^<20^	^134^	^-^

## Discussion

ITG, first described by Schwartz and Lewis in 1980, occurs in approximately 0.05-0.1% of native renal biopsies [1-3]. These tactoids are microtubular immune deposits arranged in parallel with hollow cores, typically IgG1/IgG2 dominant. They are often monoclonal (associated with hematological disorders) or polyclonal (linked with infection/autoimmunity).

Two large retrospective studies by Nasr et al. (2012, 2021) and one by Javaugue et al. form the foundation of current understanding [4,5,7]. The 2012 Nasr study provided early insights into the clinicopathologic and proteomic features of ITG [4], while the 2021 study revealed that monoclonal ITG is frequently associated with B-cell malignancies and benefits from clone-targeted therapy [5]. In contrast, polyclonal cases, such as the one described here, are typically associated with chronic infections or immune dysregulation. Only one-fourth of these cases are associated with hematological disorders.

Our patient had no hematological disorder despite thorough workup. Her polyclonal ITG likely resulted from long-standing HIV infection and HCV seropositivity. The association between HCV and ITG was first systematically described by Markowitz et al., who reported six cases of fibrillary glomerular disease (including two ITG cases) in HCV-infected patients [10]. Similarly, the association with HIV was established by Haas et al., who described three cases of fibrillary/immunotactoid glomerulonephritis in HIV-positive patients [8]. Description of ITG in patients with HIV with and without HCV and hepatitis B virus (HBV) have been described in the literature as well [11].

Antiretroviral therapy is the foundation of the management of ITG associated with HIV. Robust evidence supporting the use of immunosuppression is lacking in this subgroup of patients. Our patient was treated with antiretroviral therapy with renin-angiotensin-aldosterone system inhibitors (RAASi) followed by addition of SGLT2 inhibitors. This multidisciplinary treatment approach led to a reduction in her proteinuria.

This represents a novel therapeutic approach in ITG management, avoiding the risks associated with immunosuppression in immunocompromised patients.

## Conclusions

Our case represents an occurrence of immunotactoid glomerulopathy in a patient with concurrent HIV infection and anti-HCV antibody positivity. Our findings demonstrate that polyclonal ITG in immunocompromised patients can be effectively managed without traditional immunosuppressive therapy through a targeted approach combining optimized antiretroviral therapy and nephroprotective agents.

The successful use of SGLT2 inhibitors in this clinical context offers a novel therapeutic strategy that avoids the substantial risks associated with immunosuppression in vulnerable populations. The significant reduction in proteinuria from 7,834 mg/gm to 1,543 mg/gm, coupled with preserved renal function throughout the treatment course, supports this conservative management approach. However, a longer longitudinal follow-up is needed to establish the validity of the treatment strategy.

This case highlights the importance of considering ITG in the differential diagnosis of nephrotic syndrome in patients with chronic viral infections and underscores the need for individualized treatment strategies based on underlying immune status. The favorable clinical outcomes achieved in our patient may inform future management approaches for similar cases and warrant further investigation in larger cohorts of immunocompromised patients with ITG.
